# Effects of moderate-intensity exercise on social health and physical and mental health of methamphetamine-dependent individuals: A randomized controlled trial

**DOI:** 10.3389/fpsyt.2022.997960

**Published:** 2022-09-23

**Authors:** Jisheng Xu, Zhicheng Zhu, Xin Liang, Qiuyue Huang, TianZhen Zheng, Xue Li

**Affiliations:** School of Sports Medicine and Health, Chengdu Sport University, Chengdu, China

**Keywords:** methamphetamine, exercise, social health, addiction, treatment

## Abstract

**Objective:**

Methamphetamine (MA)-dependent individuals’ health problems are widespread and need to be solved urgently. Exercise is considered a potential treatment for MA dependents. The study aimed to determine the effects of a 12-week aerobic exercise on the social, physical, and mental health of MA-dependent individuals.

**Materials and methods:**

Sixty MA-dependent individuals were randomly assigned into two groups. Subjects in the exercise group (*n* = 30) received an exercise intervention five days a week for 60 min each for 12 weeks. Subjects in the control group (*n* = 30) received regular corrective rehabilitation without exercise in the same setting. Outcome measures, including questionnaires [quality of life scale for drug addiction (QOL-DA), self-rating anxiety scale (SAS), self-rating depression scale (SDS), and Pittsburgh sleep quality index (PSQI)] and physical fitness, were arranged the day before the start of the intervention and the day after the end of the intervention. Two-factor repeated measures ANOVA was used to compare the treatment differences between the two groups.

**Results:**

After 12 weeks of the intervention period, social health was significantly improved in the exercise group (*P* < 0.01), and there was a statistically significant difference in mental health scores between exercise group and control group, with a greater impact in exercise group.(Psychology: *P* < 0.01; SAS: *P* < 0.01; SDS: *P* < 0.01; PSQI: *P* < 0.01), physical health improved in the exercise group, physiology (*P* < 0.01), symptom (*P* < 0.01), heart rate (*P* < 0.01), systolic blood pressure (*P* < 0.01), systolic blood pressure (*P* < 0.01), vital capacity (*P* < 0.05), grip (*P* < 0.01), vertical jump (*P* < 0.001), sit and reach (*P* < 0.01), 50-meter run (*P* < 0.01), and reaction time (*P* < 0.01).

**Conclusion:**

Aerobic exercise intervention is an effective treatment for MA-dependent individuals, and the 12-week intervention improved the social, physical, and mental health of MA-dependent individuals. We recommend that future studies focus more on drug-dependent individuals’ overall health status rather than just relapse.**Clinical trial registration:** [https://www.chictr.org.cn/hvshowproject.aspx?id=131048], identifier [ChiCTR2200055348].

## Introduction

Health is defined as “a state of complete physical, mental and social well-being and not merely the absence of disease or infirmity” (1). Among them, social health is considered a dynamic balance between opportunities and limitations; the constant changes in life, society, and the environment bring endless restrictions and challenge people’s ability to adapt to this state (1). Methamphetamine (MA) is a powerful, illicit psychostimulant (2, 3) that causes some degree of impairment to social, physical, and mental health when ingested. The clinical response to acute MA use is characterized by euphoria, reduced fatigue, and social activation (4, 5), leading to a false “ideal state of health.” However, the legacy of mental impairment from substance use can eventually lead to impaired social functioning and even isolation in MA abusers (6). At the same time, poor social competence will reinforce addictive behaviors, including MA (7, 8), creating a vicious cycle, whereas positive social interactions can prevent drug addiction to some extent (9). Furthermore, the damage to physical health from MA abuse is reflected in the increasing deterioration of physical function, cardiorespiratory and cardiovascular function, and all of its related physical indicators. Long-term use of MA has deleterious effects on motor function and muscles (10), causes excessive pulmonary stress (11), increases vascular damage, leads to persistent vascular dysfunction, and accelerates atherosclerosis (12–14). Finally, among psychological conditions, depression and anxiety are the most common disorders (15), and depression has been recognized as a hallmark of MA withdrawal symptoms (16), which may be because of abnormalities in monoamine neurotransmitter pathways, such as dopamine, serotonin, and norepinephrine, as a result of MA abuse (17, 18). Sleep difficulties during MA withdrawal are also a relatively rare symptom compared with other drugs of abuse (4). The poor impact of MA abuse on social, physical, and mental health leads to high relapse rates (19, 20) and is also accompanied by higher suicide attempts (21, 22). New evidence suggests that methamphetamine abuse may become the next substance abuse crisis worldwide (23, 24).

Physical exercise is considered a potential treatment for MA addiction (25–27). And numerous mechanisms have been proposed to explain why exercise can improve the health of MA-dependent individuals, including the improvement of the nervous system, the improvement of the inhibitory control ability, the recovery of the monoaminergic system, the recovery of the blood-brain barrier, and the remodeling of cortical function (28–32). Indeed, clinical trials indicate long-term aerobic exercise is effective in reducing depressive and anxious emotional states and improving physical health and quality of life in MA-dependent individuals (33). Aerobic exercise improves physical health indicators, such as lung capacity, grip strength, and standing on one foot with eyes closed, in MA-dependent individuals (34, 35). Meanwhile, a recent meta-analysis suggested that exercise showed better therapeutic effects on the physical health of ATS-dependent individuals (35). In addition, exercise training three times a week can enhance heart rate variability in MA-dependent individuals by increasing the balance between vagal regulation and autonomic control (36), intending to protect cardiovascular function in MA-dependent individuals. A recent systematic review demonstrated that exercise can be effective in regulating addiction in drug-dependent individuals while serving as a stress management tool to improve their mental health (37). Level II evidence suggests that exercise is effective in reducing anxiety and depression in MA-dependent individuals (38). Also, clinical trials have found significant improvements in depression and anxiety symptoms in newly admitted MA-dependent individuals who received an eight-week exercise intervention, and the effects continued to be significant over time (39). For social health, research has found that physical activity can facilitate the establishment of positive social contact among substance use disorders (40). In addition, group-based exercise has been shown to have significant benefits on the social functioning of drug-dependent individuals (41). There is a paucity of research on the social health of MA dependents with exercise therapy and a lack of evaluation of the effects of exercise therapy on them from an overall health perspective.

Therefore, the present study attempted to assess the effects of moderate-intensity aerobic exercise on the social, physical, and mental health of MA-dependent individuals from a health perspective. Also, expand the research direction in this field. Based on the existing studies and literature, we hypothesized that moderate-intensity aerobic exercise could improve the social, physical, and mental health of MA-dependent individuals.

## Materials and methods

### Study design

This study used a single-blind (assessor-blind), randomized, clinical, parallel-group intervention. The recruitment of participants and the conduct of the trial were conducted at the Ziyang Drug Rehabilitation Institute in Sichuan Province. Open recruitment was adopted to recruit willing subjects by conducting a centralized presentation to drug addicts in the institute. The study was approved by the Ethics Committee of the Chengdu Sports University [Grant No. (2021) 14] and all experimental procedures followed the Declaration of Helsinki, a guideline for human medical research (42). All participants provided written informed consent. The current study has been registered on the platform of the China Trial Registration Center (Registration number: ChiCTR2200055348).

### Sample size

The sample size required for the current trial was calculated by PASS 15.0 statistical software. Based on a previous report (43), using an independent samples *t*-test, set at α = 0.05 (two-sided) and β = 0.1, we estimated that a sample size of 22 cases per group was required after allocation, and considering a 15% loss to follow-up rate of study participants, a minimum of 26 participants per group should be secured. Ultimately 30 participants were included in each group in this study.

### Participants

**FIGURE 1:**
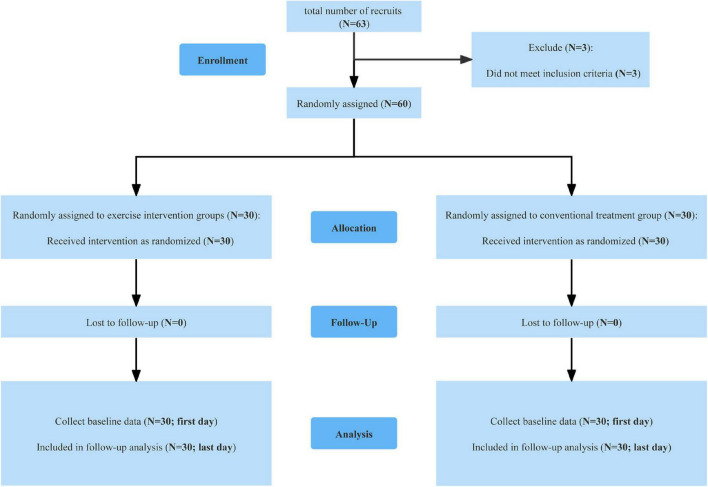
Flow-chart of the experimental procedure.

A total of 63 MA-dependent individuals were recruited for this study at Ziyang Drug Rehabilitation Center in Sichuan Province, of which three subjects did not meet the inclusion criteria. A total of 60 validated MA-dependent individuals were recruited after screening by strict inclusion and exclusion criteria. General information, including age, height, weight, occupation, marital status, years of drug use, and average dose of drug use, were collected from the study subjects who met the inclusion criteria. The subjects were randomly assigned to the exercise intervention group (*n* = 30) and the conventional treatment group (*n* = 30) by another researcher not involved in this study using the random number table method. In brief, 60 participants were assigned numbers (1, 2, 3.60) recorded in an Excel sheet, and then 60 random numbers were generated, after which they were sorted in ascending order, with the top 30 being included in the exercise group and the bottom 30 in the control group. The flowchart of the experimental procedure is shown in Figure 1.

Inclusion and exclusion criteria

1. Inclusion criteria: (1) between 18–55 years old and eligible for the fifth edition of the Diagnostic and Statistical Manual of Mental Disorders (DSM-?) methamphetamine substance dependence, (2) with elementary school education or above, (3) assessed to be free of exercise risk, (4) can guarantee more than six months of recovery time, (5) voluntarily participated and signed an informed consent form.

2. Exclusion criteria: (1) with infectious diseases, such as hepatitis and the human immunodeficiency virus (HIV) and serious untreated trauma; (2) with recent neurological injuries, such as cranial brain injury and spinal cord injury, neurodegenerative diseases, or serious psychiatric diseases; (3) suffering from serious organic diseases; (4) with addictive behaviors other than methamphetamine addiction; (5) having ingested drugs, such as methadone, for addiction treatment within the last four months.

### Exercise intervention

The exercise intervention program was moderate-intensity aerobic exercise according to Physical Activity Guidelines for Chinese (44). Based on dose-response studies, moderate-intensity exercise appears to be the optimal intensity for treating substance use disorders (45–47). The exercise group received moderate-intensity aerobic exercise for 1 h 5 times per week for 12 weeks. The exercise program includes 10 min of warm-up training (jogging, warm-up activities or dynamic stretching), 30 min of aerobic training (cycling, jogging or calisthenics), and 20 min of stretching (static stretching for the training area) each day. The exercise program was performed by two master’s degree students in kinesiology, and the participants’ real-time heart rate was monitored by a polar heart rate sensor (BHT Gofit 3.0), which was used to control the intensity of exercise during the one-week adaptation period (65–70% HRmax; HRmax = 206.9–0.67*age), and the heart rate was controlled at (70–75% HRmax) (48). To ensure that all subjects received the same exercise intervention, one interventionist led the training while the other supervised the subject’s movements to meet the standards and monitored the subject’s heart rate in real time via a mobile device. The control group received regular corrective rehabilitation treatment in a rehabilitation institution, including educational correction, group counseling, etc. (the matrix model was not adopted), and did not perform any exercise and was scheduled to perform at the same time as the exercise group. The rest of the time, the two groups lived the same lives.

### Outcome

The primary outcome was the social health of the subjects, which is measured with the quality of life scale for drug addiction (QOL-DA) (49). Quality of life covers the whole spectrum of health (physical, psychological, social, etc.) and allows a comprehensive evaluation of the impact of the disease and its treatment on the physical, psychological and social aspects of the patient’s life. Health-related quality of life has also been applied to measure the health status of substance use disorders. In addition, QOL-DA was developed in 1997 specifically for drug-dependent patients in China. Four measurement dimensions, social, psychological, symptomatic, and somatic, consist of 40 items, of which the social health dimension will be used as the primary outcome. Psychological, symptom, and physical dimensions will be used as secondary outcomes along with mental and physical health.

Mental health measures include subjective depression, subjective anxiety, and sleep quality. Self-rating depression scale (SDS) (50) and self-rating anxiety scale (SAS) (51) were separately used to assess subjects’ depression and anxiety. SDS consists of 20 items, of which items 2, 5, 6, 11, 12, 14, 16, 17, 18, and 20 are scored in reverse, and the rest are scored positively. Score*1.25 is used to obtain the standard score. The greater the standard score, the more severe the depression. Similar to the SDS, the SAS also consists of 20 items (reverse scoring items: 5, 9, 13, 17, and 19) and is also assessed by a standard score. The higher the standard score, the more anxious the subject. Pittsburgh Sleep Quality Index (PSQI) (52) is used to assess sleep quality. It consists of 19 individual assessment items and is divided into seven components: sleep quality, sleep onset time, sleep time, sleep efficiency, sleep disorders, hypnotic drugs, and daytime dysfunction (53).

The test of physical health includes heart rate, blood pressure, vital capacity, waist-to-hip ratio (WHR) (waist and hip circumference), body mass index (BMI) (height and weight), grip, vertical jump, sit-and-reach, 50-m running, and reaction time. The test equipment adopts the national sports equipment (heart rate and blood pressure machine, spirometer, soft ruler, grip strength device, longitudinal jumping device, sit-and-reach device, reaction time measuring device). The general administration specifies the equipment and strictly follows the physical test standards.

Outcome measurements were scheduled the day before and after the experimental intervention and were measured by professional researchers at the Chengdu Sport University.

### Statistical methods

Data were analyzed by two independent researchers, and all statistical analyses were performed using IBM SPSS for Windows 26.0. Data are presented as mean (standard deviation, SD), median (interquartile range, IQR), or count (%). Differences in clinical baseline characteristics and outcome measures between the two groups of participants were measured using the chi-square test of homogeneity (categorical variables), independent samples *t*-tests (normally distributed continuous variables), and non-parametric independent samples *t*-tests (non-normally distributed continuous variable). Outcome parameters for social, physical, and mental health were assessed using 2 (group: exercise and control) × 2 (time: pre-test and post-test) repeated measures ANOVA. When outcomes with significant interaction, examine by analyzing the simple effects, otherwise consider the main effects. Statistical significance was established at *P* < 0.05.

## Results

### Baseline characteristics of participants

The demographic data showed no statistically significant difference in age, height, weight, BMI, work status, marital status, education level, years of drug use, average dose and frequency of MA between subjects in groups of exercise and control (*P* > 0.05) (Table 1).

**TABLE 1:** Baseline characteristics of participants.

Characteristic	Exercise group	Control group	*P*-value
	(*n* = 30)	(*n* = 30)	
Mean age (SD), y	31.30 (3.86)	29.50 (4.59)	0.10
Mean height (SD), cm	167.82 (4.93)	170.02 (4.12)	0.07
Mean body weight (SD), kg	67.54 (7.67)	69.08 (6.77)	0.42
Mean BMI (IQR), kg/m^2^^a^	24.22 (22.31, 25.16)	22.74 (22.25, 25.76)	0.66
Occupation, n (%)			0.61
Employed/Self-employed	15 (50)	17 (57)	
Unemployed	15 (50)	13 (43)	
Marital status, n (%)			0.50
Married	9 (30)	8 (27)	
Single	14 (47)	18 (60)	
Divorced	7 (23)	4 (13)	
Education (IQR)^b^	2 (1, 3)	2 (1,3)	0.50
Years of drug use (IQR)^c^	4 (3, 4)	4 (3, 4)	0.57
Average dose of drug use (IQR)^d^	3 (2, 3)	2 (2, 3)	0.09
Frequency of drug use (IQR)^e^	2 (1, 3)	2 (1, 3)	0.79

Data were presented as the mean ± (SD), median (IQR), or count (%).

^a^BMI = body mass index; height (m)/body weight (kg)^2^.

^b^Unit for Education: 1 = primary school, educated for 6 years; 2 = junior high school, educated for 9 years; 3 = senior high school, educated for 12 years; 4 = college, educated for 16 years.

^c^Years of drug use are defined as from the time of the first drug use to the last drug use: 1 = less than 1 year, 2 = 1–3 years, 3 = 4–9 years, 4 = 10–15 years, and 5 = more than 15 years.

^d^Average dose of drug use was obtained through subject recalls or exchanges with drug rehabilitation authorities: 1 = less than 0.1 g at a time, 2 = 0.1–0.3 g at a time, 3 = 0.1–1.0 g at a time, 4 = more than 1.0 g at a time. ^e^Frequency of drug use: 1 = once or less per week; 2 = 2–5 times a week; 3 = once a day or more.

### Changes in the social health of mA-dependent individuals after exercise intervention

**FIGURE 2:**
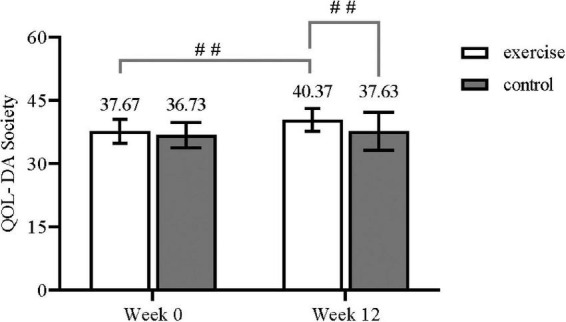
QOL-DA society in each group (^##^ Displayed as *P*<0.01).

The results showed a strong statistically significant trend in the time and group interaction effect for social health (*F*_(1, 29)_ = 4.17, *P* = 0.05, η^2^ = 0.126). Based on this result, we then conducted a simple effects analysis and found that in the exercise group, the post-intervention significantly increased compared to the pre-intervention (*F*_(1, 29)_ = 24.94, *P* < 0.01, η ^2^ = 0.462). And in the control group, there was no statistical difference before and after the intervention (*F*_(1, 29)_ = 2.73, *P* = 0.11, η^2^ = 0.086). In addition, the scores on the post-test of the exercise group were also higher than those on the post-test of the control group and were statistically different (*F*_(1, 29)_ = 10.49, *P* < 0.01, η^2^ = 0.266) (Figure 2 and Table 2).

**TABLE 2:** Primary outcomes: Social health.

Outcome	Exercise group (*n* = 30)		Control group (*n* = 30)		Within group *F*-value	Between group *F-*value	Time*group interaction *F*-value
					
	Week 0	Week 12	Week 0	Week 12			
QOL-DA Society	37.67 (2.88)	40.37 (2.75)	36.73 (3.02)	37.63 (4.50)	32.29**^##^**	18.79**^##^**	4.17

Data were analyzed with repeated measures ANOVA and presented as the mean ± (SD).

**^##^**Displayed as *P*<0.01.

### Changes in the mental health of mA-dependent individuals after exercise intervention

The results showed that there was a time and group interaction effect for QOL-DA Psychology (*F*_(1, 29)_ = 4.80, *P* < 0.05, η^2^ = 0.142), SAS (*F*_(1, 29)_ = 20.85, *P* < 0.01, η ^2^ = 0.418), SDS(*F*_(1, 29)_ = 58.67, *P* < 0.01, η^2^ = 0.669), and PSQI (*F*_(1, 29)_ = 12.88, *P* < 0.01, η^2^ = 0.308). Further simple effects analysis revealed that in the exercise group, there were statistically significant differences between scores for each outcome before and after the intervention [QOL-DA Psychology: (*F*_(1, 29)_ = 7.01, *P* < 0.05, η^2^ = 0.195); SAS: (*F*_(1, 29)_ = 72.38, *P* < 0.01, η^2^ = 0.714; SDS): (*F*_(1, 29)_ = 104.30, *P* < 0.01, η^2^ = 0.782; PSQI): (*F*_(1, 29)_ = 20.90, *P* < 0.01, η^2^ = 0.418)], and the effects were focused on the post-intervention period. In the control group, the post-intervention SAS scores were more severe and statistically different (*F*_(1, 29)_ = 4.76, *P* < 0.05, η^2^ = 0.141). In addition, the remaining three indicators were not statistically different before and after the intervention (QOL-DA Psychology: *F*_(1, 29)_ = 0.001, *P* = 0.97, η^2^ < 0.001; SDS: *F*_(1, 29)_ = 0.18, *P* = 0.68, η^2^ = 0.006); PSQI: (*F*_(1, 29)_ = 1.40, *P* = 0.25, η^2^ = 0.046). Also, after the intervention, the QOL-DA Psychology scores of the exercise group were significantly higher than those of the control group (*F*_(1, 29)_ = 19.98, *P* < 0.01, η^2^ = 0.408), while the SDS, SAS and PSQI scores were lower than those of the control group [SAS: (*F*_(1, 29)_ = 59.59, *P* < 0.01, η^2^ = 0.673); SDS: (*F*_(1, 29)_ = 91.43, *P* < 0.01, η^2^ = 0.759); PSQI: (*F*_(1, 29)_ = 27.96, *P* < 0.01, η^2^ = 0.491)] (Table 3).

**TABLE 3:** Secondary outcomes: Mental health.

Outcome	Exercise group (*n* = 30)		Control group (*n* = 30)		Within group *F*-value	Between group *F-*value	Time*group interaction *F*-value
					
	Week 0	Week 12	Week 0	Week 12			
QOL-DA Psychology	37.77 (3.95)	40.20 (3.24)	37.03 (5.40)	37.07 (5.00)	3.05	7.26^#^	4.80^#^
SAS^a^	44.43 (7.65)	31.77 (3.89)	45.77 (5.64)	42.13 (6.24)	43.80^##^	36.02^##^	20.85^##^
SDS^b^	53.20 (5.12)	39.37 (6.09)	54.80 (8.95)	54.07 (6.12)	31.42^##^	77.82^##^	58.67^##^
PSQI^c^	6.83 (2.36)	4.57 (1.38)	6.27 (2.08)	6.83 (1.53)	8.84^##^	4.25^#^	12.88^##^

Data were analyzed with repeated measures ANOVA and presented as the mean ± (SD).

**^#^**Displayed as *P*<0.05.

**^##^**Displayed as *P*<0.01.

^a^SAS = Self-rating anxiety scale.

^b^SDS = Self-rating depression scale.

^c^PSQI = Pittsburgh sleep quality index.

### Changes in the physical health of mA-dependent individuals after exercise intervention

The results showed a time and group interaction for five physical health outcomes, including QOL-DA Symptom (*F*_(1, 29)_ = 47.54, *P* < 0.01, η^2^ = 0.621), QOL-DA Physiology (*F*_(1, 29)_ = 17.857, *P* < 0.01, η^2^ = 0.381), vertical jump (*F*_(1, 29)_ = 8.82, *P* < 0.01, η^2^ = 0.233), sit-and-reach (*F*_(1, 29)_ = 12.42, *P* < 0.01, η^2^ = 0.30), 50-m running (*F*_(1, 29)_ = 11.87, *P* < 0.01, η^2^ = 0.290). A simple effects analysis of these outcomes found that in the exercise group, QOL-DA Symptom (*F*_(1, 29)_ = 8.61, *P* < 0.01, η^2^ = 0.229), vertical jump (*F*_(1, 29)_ = 20.38, *P* < 0.01, η^2^ = 0.413) and sit-and-reach (*F*_(1, 29)_ = 5.78, *P* < 0.05, η^2^ = 0.166) were statistically different before and after the intervention. In contrast, QOL-DA Physiology (*F*_(1, 29)_ = 0.18, *P* = 0.68, η^2^ = 0.006) and 50-m running (*F*_(1, 29)_ = 0.61, *P* = 0.44, η^2^ = 0.020) were not statistically different before and after the intervention. Vertical jump (*F*_(1, 29)_ = 0.13, *P* = 0.721, η^2^ = 0.004) in the control group was not statistically different, whereas QOL-DA Symptom (*F*_(1, 29)_ = 49.66, *P* < 0.01, η^2^ = 0.631), QOL-DA Physiology (*F*_(1, 29)_ = 43.21, *P* < 0.01, η^2^ = 0.598), sit-and-reach (*F*_(1, 29)_ = 6.06, *P* < 0.05, η^2^ = 0.173) and 50-meter running (*F*_(1, 29)_ = 12.19, *P* < 0.05, η^2^ = 0.296) were statistically different. In addition, QOL-DA Physiology (*F*_(1, 29)_ = 22.80, *P* < 0.01, η^2^ = 0.440), QOL-DA Symptom (*F*_(1, 29)_ = 32.78, *P* < 0.01, η^2^ = 0.531), vertical jump (*F*_(1, 29)_ = 93.41, *P* < 0.01, η^2^ = 0.763), sit-and-reach (*F*_(1, 29)_ = 8.68, *P* < 0.01, η^2^ = 0.230), and 50-meter running (*F*_(1, 29)_ = 228.85, *P* < 0.01, η^2^ = 0.890) were significantly higher in the exercise group than in the control group after the intervention.

Within-group factors after 12 weeks of exercise intervention were statistically different in heart rate (*F*_(1, 29)_ = 27.92, *P* < 0.01, η^2^ = 0.490), systolic blood pressure (*F*_(1, 29)_ = 8.59, *P* < 0.01, η^2^ = 0.228), vital capacity (*F*_(1, 29)_ = 7.29, *P* < 0.05, η^2^ = 0.201), grip (*F*_(1, 29)_ = 120.80, *P* < 0.01, η^2^ = 0.806), and reaction time (*F*_(1, 29)_ = 58.08, *P* < 0.01, η^2^ = 0.667). Statistical differences were found in systolic blood pressure (*F*_(1, 29)_ = 5.72, *P* < 0.05, η^2^ = 0.165) and WHR (*F*_(1, 29)_ = 6.73, *P* < 0.05, η^2^ = 0.188) among the between-group factors (Table 4).

**TABLE 4:** Secondary outcomes: Physical health.

Outcome	Exercise group (*n* = 30)		Control group (*n* = 30)		Within group *F*-value	Between group *F-*value	Time*group interaction *F*-value
					
	Week 0	Week 12	Week 0	Week 12			
QOL-DA Symptom	49.77 (3.57)	52.27 (3.43)	51.40 (1.89)	46.40 (4.35)	4.90**^#^**	8.17**^##^**	47.54**^##^**
QOL-DA Physiology	36.47 (3.48)	36.13 (3.12)	37.13 (2.93)	31.80 (3.75)	27.37**^##^**	7.63**^##^**	17.86**^##^**
Heart Rate (bpm)	82.37 (12.92)	73.13 (11.68)	79.33 (11.29)	73.17 (10.62)	27.92**^##^**	0.32	0.38
Systolic Blood Pressure (mmHg)	124.33 (8.40)	115.03 (16.07)	125.53 (10.85)	123.03 (11.06)	8.59**^##^**	5.72**^#^**	1.98
Diastolic Blood Pressure (mmHg)	75.77 (8.20)	73.13 (14.21)	75.27 (7.33)	76.33 (7.37)	0.17	0.69	1.11
Vital Capacity (ml)	3442.60 (554.20)	3669.10 (504.88)	3502.50 (644.60)	3762.20 (485.93)	7.29**^#^**	0.62	0.03
WHR^a^	0.88 (0.06)	0.86 (0.05)	0.89 (0.05)	0.89 (0.04)	2.32	6.73**^#^**	1.25
BMI^b^	23.97 (2.41)	24.14 (2.35)	23.93 (2.54)	24.08 (2.78)	2.09	0.02	0.01
Grip (kg)	38.22 (6.93)	47.96 (7.82)	37.72 (7.99)	45.86 (5.16)	120.80**^##^**	2.54	1.25
Vertical Jump (cm)	35.15 (7.27)	39.83 (5.75)	35.41 (7.66)	34.82 (4.12)	3.72	4.94**^#^**	8.82**^##^**
Sit-and-Reach (cm)	11.22 (9.62)	14.18 (6.96)	11.87 (6.44)	9.84 (4.67)	0.37	1.19	12.42**^##^**
50-Meter Running (s)	8.67 (0.64)	8.55 (0.65)	8.44 (0.58)	8.93 (0.54)	2.74	0.70	11.87**^##^**
Reaction Time (s)	0.54 (0.09)	0.43 (0.04)	0.55 (0.10)	0.48 (0.05)	58.08**^##^**	2.34	3.85

Data were analyzed with repeated measures ANOVA and presented as the mean ± (SD).

**^#^**Displayed as *P*<0.05.

**^##^**Displayed as *P*<0.01.

^a^WHR = Waist-to-Hip Ratio; waist (cm)/hip (cm).

^b^BMI = Body Mass Index; body weight (kg)/height (m)^2^.

## Discussion

Clinical symptoms of MA abuse include social health problems caused by reduced social adaptation (6, 54). Mental health problems are caused by depression, anxiety (23), and less sleep quality (55). Physical health problems are caused by motor dysfunction (10, 56) and impaired cardiovascular function (5). The pain caused by these complex and varied symptoms can lead to uncontrollable drug relapse (19, 57), forming a vicious circle. In this study, we looked at the complete health status of MA dependents and focused on their social health indicators. This study provides evidence on the impact of a 12-week moderate-to-intensity aerobic exercise intervention on the social, physical, and mental health of MA dependents. Statistically, subjects in the exercise group had better social, mental, and physical health than those in the usual care group, and no adverse effects were reported throughout the trial.

As found in previous studies, in the area of drug abuse, adverse social activities induce a strong motivation to continue seeking drugs (58, 59) and enhance drug relapse (60, 61). Individuals with concurrent social health problems have a higher risk of death (62). While MA abusers may have persistently high levels of psychological distress and hostility that are detrimental to their social interactions, health concerns in the social sphere of MA dependents are worthwhile (63). However, studies on exercise therapy for the social health of MA dependents are relatively few. One study found that low to moderate intensity physical and mental exercise for 3 months could positively impact social functioning in individuals with substance use disorders (43). In addition, acute exercise interventions, either 1 h of aerobic exercise alone or 1 h of strength combined with aerobic exercise, can improve social health in amphetamine addicts (64). Their findings are consistent with the present study’s; that is, physical exercise improves the social health status of MA dependents, which may be because exercise intervention repairs damaged brain regions or nervous systems in MA abusers (32). This view that exercise can reduce addiction is widely accepted (65, 66). The functional brain regions responsible for processing social inclusion and exclusion are mainly located in the insula (67, 68), and drug abuse will directly damage the insula’s neural structures. At the same time, MA abuse leads to an imbalance in the dopaminergic system, in which type 1 dopamine receptor signaling in the ventral tegmental area mediates complex social behavior (69) and the availability of striatal dopamine D2/3 receptors correlates with subjects. The correlation between social status and perceived social support is positive (70). As a treatment for drug addicts, physical exercise’s effectiveness on social health may involve repairing or protecting neural structures such as the insula and dopamine system. (71–73), which may explain why exercise can improve the social well-being of MA-dependent individuals.

The scores of psychological symptoms, self-rating depression, self-rating anxiety, and sleep quality index in the exercise group were significantly improved after 12 weeks of exercise intervention. These results did not change in the control group, and the health scores improved over time. This is similar to a previous study that eight weeks of exercise training improved symptoms of depression and anxiety in MA-dependent individuals (39). It is worth noting that the effectiveness of exercise in improving sleep quality (74, 75), although constantly proven, is rarely mentioned in MA dependents (76). Our findings support the use of 12 weeks of moderate-intensity aerobic exercise as an effective prescription for improving mental health and enhancing sleep quality in MA-dependent individuals. In terms of physical health, the 12-week exercise intervention reduced the withdrawal symptoms of MA-dependent individuals and improved their physical function. The cardiovascular benefits were also consistent with previous studies. Exercise lowered blood pressure in MA-dependent individuals. Physical, strength, flexibility, speed, and agility quality have improved, which is consistent with previous research results (39, 77); that is, MA-dependent individuals who participate in sports will have better physical health and psychological effects (43).

The study found no adverse events during the intervention, and no subjects dropped out of the trial, suggesting that moderate-intensity aerobic exercise can be safely used in MA-dependent individuals to restore their health. And, the effectiveness of the exercise program in this study (12 weeks of moderate-intensity aerobic exercise for 1 h, 5 times per week) could inform targeted exercise prescriptions for drug-dependent individuals in future studies. However, this study still has several limitations. First, the sample size of this study is small. Second, the selected participants were all male; thus, the effect of gender could not be assessed. Finally, during the implementation period of the trial, the subjects were not banned from tobacco use, which may have potential effects on the subjects’ cardiorespiratory fitness. Therefore, in future work, while expanding the sample size, more female subjects should be included in the trial research to obtain more comprehensive evidence. Despite the study limitations, the data from this study can provide preliminary evidence that exercise improves the health of MA-dependent individuals, and the overall health of special populations should be given focus.

## Conclusion

This study shows that moderate-intensity aerobic exercise intervention is an effective treatment for MA-dependent individuals and that 12 weeks of intervention improved social well-being, depression, anxiety, and sleep in MA-dependent individuals. The resulting mental health also enhanced physical health, including systolic blood pressure, WHR, vertical jump, seated forward bend, 50-m run, and reaction time. Future studies should pay more attention to the overall health status of drug-dependent individuals rather than just their relapse.

## Data availability statement

The raw data supporting the conclusions of this article will be made available by the authors, without undue reservation.

## Ethics statement

The studies involving human participants were reviewed and approved by the Ethics Committee of Chengdu Sports University. The patients/participants provided their written informed consent to participate in this study.

## Author contributions

JX and ZZ: methodology, investigation, and writing – original draft. JX and XLi: conceptualization and funding acquisition. XLi: resources, project administration, supervision, and writing – review and editing. XLia: validation and data curation. QH and TZ: visualization and formal analysis. All authors have read and agreed to the published version of the manuscript.
